# A Prothrombotic Score Based on Genetic Polymorphisms of the Hemostatic System Differs in Patients with Ischemic Stroke, Myocardial Infarction, or Peripheral Arterial Occlusive Disease

**DOI:** 10.3389/fcvm.2017.00039

**Published:** 2017-06-09

**Authors:** Juliane Herm, Berthold Hoppe, Bob Siegerink, Christian H. Nolte, Jürgen Koscielny, Karl Georg Haeusler

**Affiliations:** ^1^Department of Neurology, Charité – Universitätsmedizin Berlin, Berlin, Germany; ^2^Center for Stroke Research Berlin, Charité – Universitätsmedizin Berlin, Berlin, Germany; ^3^Institute of Laboratory Medicine and Pathobiochemistry, Charité – Universitätsmedizin Berlin, Berlin, Germany; ^4^Institute of Laboratory Medicine, Unfallkrankenhaus Berlin, Berlin, Germany; ^5^Berlin Institute of Health, Berlin, Germany; ^6^Institute of Transfusion Medicine, Charité – Universitätsmedizin Berlin, Berlin, Germany

**Keywords:** thrombophilia, ischemic stroke, myocardial infarction, PAOD, prothrombotic state

## Abstract

**Background:**

While twin studies indicate a genetic component in arterial thrombosis such as ischemic stroke, myocardial infarction (MI), or peripheral arterial occlusive disease (PAOD), the clinical relevance of hemostatic polymorphisms in arterial thrombosis is a matter of debate.

**Methods:**

We analyzed the prevalence of 13 hemostatic polymorphisms [*PAI-1, PLAT, F5* (including factor V *Leiden* and HR2 haplotype), *F2, F7, F13A, FGB, TFPI, THBD, MTHFR, ACE*, and *ITGA2*] in patients referred to a tertiary referral center. A “prothrombotic score” was calculated by dividing the number of risk-increasing polymorphisms for thrombosis minus the number of risk-lowering polymorphisms (*F7* and *F13A*) by the number of polymorphisms tested.

**Results:**

Datasets of 144 patients with prior ischemic stroke (mean age 44 ± 13 years; 65% female) were compared to 62 patients with MI or PAOD (mean age 54 ± 14 years; 47% female). The prothrombotic score was lower in MI and PAOD patients compared to stroke patients [odds ratios 2.7 (95% confidence intervals 1.1–6.2)]. Frequencies of individual polymorphisms did not differ between both groups.

**Conclusion:**

Patients with MI or PAOD had a lower burden of prothrombotic mutations compared to patients with prior stroke, indicating that a prothrombotic state might play a different role in distinct forms of arterial thrombosis.

## Introduction

Over the last years, a variety of gene polymorphisms with potential influence on the hemostatic system were described, which disturb the balance of the hemostatic system, including procoagulatory, anticoagulatory as well as fibrinolytic and antifibrinolytic pathways (1, 2). Carriership of some polymorphisms is linked to thrombophilia—an increased clotting propensity that is associated with an increased risk of venous thromboembolic events (VTE). However, whether these polymorphisms are also accompanied by an increased risk of arterial thrombosis such as stroke, myocardial infarction (MI), or peripheral arterial occlusive disease (PAOD), or a recurrence thereof, is still a matter of debate (1, 3, 4).

While the clinical relevance of factor V Leiden (*F5*) (leading to hypercoagulability due to a reduced ability of factor V variant degradation) and prothrombin (*F2*) 20210G>A mutation (leading to increased levels of prothrombin possibly due to increased pre-mRNA stability) has been extensively characterized, the effect of other so-called thrombophilic risk factors, e.g., affecting β-fibrinogen (*FGB*) or tissue-factor-pathway-inhibitor (*TFPI*) is largely unknown. For FGB, an effect on the synthesis rate of fibrinogen is assumed. Genetic predisposition is determined by multiple genetic variations. Thus, a comparison of single genetic variants in often small study populations is likely to be impaired by a varying distribution of other contributing polymorphisms, low penetrance, and low relative risks (5). Moreover, therapeutic implications of genetic subtyping are limited. Subsequently, current guidelines do not support the routine analysis of genetic polymorphisms in patients with arterial thrombosis (6, 7). Possibly, a combination or load of different genetic polymorphisms is more informative. This introduces the concept of comparing the overall prothrombotic predisposition—as previously used in venous thrombosis—in distinct cardiovascular diseases (8). Previous research indicates that hypercoagulability might play a different role in different subforms of arterial thrombosis (9). A recent meta-analysis suggests that a prothrombotic state has a greater impact on the risk of ischemic stroke compared to MI (10). Still, these results are based on factor-by-factor comparisons, ignoring that the sum of several procoagulant traits will convey a stronger clotting propensity. It is unknown to what extent a genetic score summarizing the clotting propensity would also be different for these two diseases. Therefore, we set out to determine whether such a score is differentially distributed in ischemic stroke patients compared to patients with MI or PAOD referred for thrombophilia testing.

## Materials and Methods

### Study Design and Study Population

This analysis was approved by the Ethics Committee of the Charité—Universitätsmedizin Berlin, Germany, and all patients gave informed consent for DNA isolation before genetic testing. We analyzed medical records of 144 patients with ischemic stroke and of 62 patients with either MI (*n* = 44) or PAOD (*n* = 18). These patients had been referred to the Institute of Transfusion Medicine, Charité—Universitätsmedizin Berlin, for thrombophilia testing. The following information was assessed from medical records: patients’ age, sex, cardiovascular risk factors, and history of VTE.

### Assessment of Genetic Polymorphisms

All patients in this study were referred for testing for hereditary thrombophilia, which is part of routine care performed at the Institute of Transfusion Medicine, Charité—Universitätsmedizin Berlin, Germany. Heterozygous and homozygous variants of the following gene polymorphisms were distinguished from wild type: plasminogen activator inhibitor type I (*PAI-1*) [−675 insertion/deletion (5G/4G)], tissue plasminogen activator (*PLAT*) (intron h deletion/insertion), *F5* (1691G>A) (*Leiden*) and HR2 haplotype (4070A>G), *F2* (20210G>A), *FGB* (455G>A), *TFPI* (536C>T), thrombomodulin (*THBD*) (127G>A), FVII (*F7*) (R353Q), FXIII (*F13A*) (Val34Leu), methylenetetrahydrofolate reductase (*MTHFR*) (677C>T), angiotensin converting enzyme (*ACE*) (intron 16 insertion/deletion), and glycoprotein Ia/IIa (*ITGA2*) (807C>T).

Genomic DNA was extracted from whole blood using GenoPrepTM Cartridges B and the GenoMTM-6 system (GenoVision, Vienna, Austria). DNA samples were analyzed by an amplification refractory mutation system using allele-specific primer pairs as previously described (11). All primers were synthesized by TIB Molbiol (Berlin, Germany). The polymerase chain reaction was carried out in a thermocycler (GeneAmp PCR System 9700, Applied Biosystems, Darmstadt, Germany) using the following temperature profiles: denaturation (96°C for 2 min), followed by 10 cycles (96°C for 15 s, 65°C for 60 s) and 20 cycles (96°C for 10 s, 61°C for 50 s, and 72°C for 30 s). Amplification products were separated using electrophoresis and made visible using electrophoresis and UV transillumination.

### Prothrombotic Score

To assess the overall burden of thrombophilia per patient, a prothrombotic score was calculated dividing the number of risk-increasing polymorphisms (heterozygote or homozygote) minus the number of risk-lowering polymorphisms (heterozygote or homozygote) by the number of all 13 polymorphisms tested. Variant alleles of F7 and F13A were considered to be protective. This prothrombotic score ranges theoretically between 0% (none of tested variants are prothrombotic) and 100% (all of the tested variants are prothrombotic), with the remark that only a subset of values can be observed as a maximum of 13 markers have been tested in these patient groups. The score was categorized in tertiles based on the given distribution in the stroke cohort. Overall, 19 (10.2%) patients had to be excluded from this analysis because of incomplete data (defined as less than 12 of 13 hemostatic polymorphisms tested). The excluded patients comprised of 14 stroke patients, 3 MI patients, and 2 POAD patients.

### Statistical Analysis

For categorical data, absolute and relative frequencies were computed. In case of continuous variables with nearly normal distribution, the mean, SD, minimal and maximal values, otherwise median, quartiles, minimal and maximal values were determined. The Fisher’s exact test was used to compare proportions for dichotomous outcomes between independent groups. Distribution of variant alleles was explored comparing carrier vs. non-carrier status by Fisher’s exact test. A direct comparison between the MI/PAOD group and the stroke group with regard to the prothrombotic score was performed using binary logistic regression, yielding odds ratios (OR) and corresponding confidence intervals (CI). To do this, we *a priori* categorized the risk score in tertiles, with the middle category as reference category. Nominal scaled outcomes were analyzed using the Mann–Whitney test.

## Results

### Study Cohort and Baseline Characteristics

Baseline characteristics of stroke patients as well as patients with MI or PAOD are depicted in Table 1. Stroke patients were significantly younger and more often female than MI or PAOD patients. Compared to patients with MI or PAOD, stroke patients suffered less often from diabetes, arterial hypertension, hyperlipoproteinaemia, or coronary arterial disease.

**Table 1:** Baseline data of stroke patients compared to patients with myocardial infarction (MI) or peripheral arterial occlusive disease (PAOD).

	Stroke patients (*n* = 144)	MI or PAOD patients (*n* = 62)
Age, years; mean (SD) (range)*	43.6 (13.2) (16–75)	54.4 (14.3) (24–78)
Female sex, *n* (%)*	93 (64.6)	29 (46.8)
Comorbidities, *n* (%)		
Prior ischemic stroke or TIA	9 (6.3)	1 (1.6)
Diabetes mellitus*	7 (4.9)	9 (14.5)
Arterial hypertension*	47 (32.6)	40 (64.5)
Hyperlipoproteinaemia*	50 (34.7)	35 (56.5)
Coronary artery disease	5 (3.5)	1 (5.6)^a^
Atrial fibrillation	4 (2.8)	5 (8.1)
Prior venous thrombotic event	13 (9.0)	8 (12.3)
Tobacco consumption; *n* (%)	44 (30.6)	23 (37.1)

*Data are given as *n* (%) if not stated otherwise*.

*p-Value < 0.05.

*^a^Numbers given for PAOD patients only*.

### Prothrombotic Score and Prevalence of Variant Alleles in Patients with Ischemic Stroke vs. Patients with MI or PAOD

In a tertile analysis, a low prothrombotic score (0–31.1%) was found in 22.3% (*n* = 29) of stroke patients and 36.8% (*n* = 21) of MI/PAOD [OR 2.66 (95% CI 1.14–6.21)] (Table 2), indicating that more MI/PAOD patients have a lower prothrombotic propensity then stroke patients. This difference was also observed if patients with a prior VTE were excluded from the analysis [OR 2.82 (95% CI 1.17–6.82)].

**Table 2:** Logistic regression analysis of the tertiled prothrombotic score (computed as the percentage of mutations per patient) of stroke patients with complete data sets (*n* = 130) compared to patients with either myocardial infarction (MI) or PAOD and complete data sets (*n* = 57).

Prothrombotic score (%)	Stroke *n* (%)	MI/PAOD *n* (%)	Odds ratios (95% confidence intervals)
0–31.1	29 (22.3)	21 (36.8)	2.66 (1.14–6.21)
31.2–38.5	44 (33.8)	12 (21.1)	1
38.6–100	57 (43.8)	24 (42.1)	1.54 (0.70–3.43)

Comparing the distribution of individual variant alleles in stroke patients (referred to the thrombophilia clinic) to MI/PAOD patients (referred to the same thrombophilia clinic) there was no statistically significant difference across groups (Table 3). In more detail, protective variant *F13A* Val34Leu was numerically more frequent in stroke patients than in MI/PAOD patients [44.8 vs. 33.1%; OR 1.58 (95% CI 0.85–2.94)], whereas *F7* R353Q was equally distributed [22.8 vs. 20.9%, OR 1.02 (95% CI 0.49–2.13)]. *F5* 1691G>A was found in 18.3% of stroke patients and 14.5% of MI/PAOD patients [OR 1.32 (95% CI 0.58–3.01)], and *F2* 20210G>A was present in 3.5% of stroke patients and 9.7% of MI/PAOD patients [OR 0.34 (95% CI 0.10–1.17)]. *TFPI* 536C>T as well as *THBD* 127G>A was found in one stroke patient (0.7%) and one MI/PAOD patient (1.6%). These results show that the prevalence of single polymorphisms was too low to draw strong conclusions about these individuals.

**Table 3:** Prevalence of gene polymorphisms in ischemic stroke patients (*n* = 144), patients with myocardial infarction (MI) or peripheral arterial occlusive disease (PAOD) (*n* = 62).

Polymorphism (mutation)	Group	*n*	Heterozygote % (*n*)	Homozygote % (*n*)	Odds ratios (95% confidence intervals)
*FGB* (−455G>A)	Stroke	132	37.9 (50)	2.3 (3)	1.34 (0.70–2.57)
	MI/PAOD	57	26.3 (15)	7.0 (4)	

*TFPI* (536C>T)	Stroke	142	0.7 (1)	0 (0)	^a^
	MI/PAOD	62	1.6 (1)	0 (0)	

*THBD* (127G>A)	Stroke	142	0.7 (1)	0 (0)	^a^
	MI/PAOD	62	1.6 (1)	0 (0)	

*F7* (R353Q)	Stroke	132	20.5 (27)	2.3 (3)	1.02 (0.49–2.13)
	MI/PAOD	58	17.7 (11)	3.2 (2)	

*F13A* (Val34Leu)	Stroke	143	36.4 (52)	8.4 (12)	1.58 (0.85–2.94)
	MI/PAOD	62	25.8 (16)	8.1 (5)	

*PAI-1* (−675 I/D)	Stroke	142	49.3 (70)	30.3 (43)	1.36 (0.67–2.73)
	MI/PAOD	62	38.7 (24)	35.5 (22)	

*PLAT* (intron h D/I)	Stroke	143	52.4 (75)	34.3 (49)	2.08 (0.98–4.43)
	MI/PAOD	62	54.8 (34)	21.0 (13)	

*F2* (20210G>A)	Stroke	141	3.5 (5)	0 (0)	0.34 (0.10–1.17)
	MI/PAOD	62	9.7 (6)	0 (0)	

*F5* (1691G>A)	Stroke	142	17.6 (25)	0.7 (1)	1.32 (0.58–3.01)
	MI/PAOD	62	12.9 (8)	1.6 (1)	

*F5* (4070A>G)	Stroke	141	12.1 (17)	1.4 (2)	1.05 (0.43–2.55)
	MI/PAOD	62	12.9 (8)	0 (0)	

*ACE* (intron 16 I/D)	Stroke	142	50.7 (72)	19.4 (28)	0.59 (0.29–1.22)
	MI/PAOD	62	46.8 (29)	33.9 (21)	

*MTHFR* (677C>T)	Stroke	142	41.5 (59)	10.6 (15)	1.41 (0.77–2.57)
	MI/PAOD	62	33.9 (21)	9.7 (6)	

*ITGA2* (807C>T)	Stroke	130	50.0 (65)	8.5 (11)	0.74 (0.39–1.43)
	MI/PAOD	55	50.9 (28)	14.5 (8)	

*^a^Analysis was not performed due to the low prevalence*.

## Discussion

In our study of patients referred for thrombophilia testing, the distribution of the prothrombotic propensity score differed between patients with stroke compared to those with MI or PAOD, while the individual variant alleles of this score did not differ significantly in these cohorts.

Ischemic stroke, MI, and PAOD are widespread diseases with tremendous socioeconomic impact (12, 13) and share similar cardiovascular risk factors. While MI and PAOD are (mainly) caused by atherosclerosis, stroke etiology remains undefined (“cryptogenic”) in about 25% of all stroke patients (14). Interestingly, published studies give reason to postulate that a prothrombotic state may play an important role in the pathophysiology of ischemic stroke (9, 10).

Our results show that the prothrombotic score (calculated as percentage of risk alleles per subject)—being the overall view of the prothrombotic predisposition of a patient—indeed provides different information. This approach has been used before to assess the risk of (recurrent) VTE and demonstrated a stronger association compared to individual genetic variants (5, 8).

Our results indicate that patients referred to a thrombophilia clinic because of MI/PAOD were more likely to have a low vs. normal thrombotic risk score than patients with ischemic stroke (OR of 2.7) (Table 2). This is in line with the hypothesis that there might be a different—and possibly additive—effect of the amount of prothrombotic genetic variants with regard to the risk of MI/PAOD or ischemic stroke, respectively.

These findings lead to the idea that a prothrombotic state may play a role in the pathophysiology of (cryptogenic) stroke (15). This is backed by the RATIO study, which evaluated up to 29 individual markers (including some genetic markers as *F5* 1691G>A, *F2* 20210G>A, *MTHFR* 677C>T, and others) and concluded that hypercoagulability is a risk factor of stroke but not MI in women aged <50 years (9). While our findings support this concept, our study was not designed to investigate the pathophysiology of variant forms of arterial thrombosis. Yet, our findings do show that the here proposed prothrombotic score—as a way to better assess the prothrombotic propensity instead of studying single factors—does potentially provide this information in more detail than an array of individual markers in patients with different types of arterial thrombosis. However, further studies are needed to evaluate this hypothesis.

### Limitations

Some limitations have to be taken into account in the interpretation of these results. First, the sample size is limited, as we included only patients referred to a tertiary referral center for genetic testing on the presence of thrombophilia. Thus, a potential referral bias has to be taken into account. This is a major problem of similar studies, which use population or healthy controls. However, we believe that a potential referral bias is minimized in the present analysis, as the reason for referral is assumed to be similar for both groups. Furthermore, the lower number of patients with MI or PAOD limits the power of our analysis. Moreover, combining MI and PAOD patients for analysis might have introduced bias as one of the two combined groups might have a different distribution. However, this bias would dilute any true association, rendering the reported odds ratio of 2.7 an underestimation of the real association. Moreover, stroke is a heterogeneous disease, and a prothrombotic state may play a stronger role in (young) stroke patients without or with just a few established cardiovascular risk factors. Of note, stroke patients referred for thrombophilia testing do not represent the overall cohort of stroke patients. As follow-up data were not available, we cannot answer the question whether risk assessment by routine genetic testing can reduce recurrence of arterial thrombosis. Finally, by reporting a wide variety of gene polymorphisms, not all polymorphisms were analyzed in every single patient with stroke, MI, or PAOD, respectively.

## Conclusion

The bigger picture of prothrombotic predisposition needs to be assessed instead of focusing on individual variant alleles. Our data indicate a different impact of hereditary thrombophilia on the risk of ischemic stroke compared to the risk of MI or PAOD. Large studies in well-defined cohorts of stroke, MI, and PAOD patients are needed to better understand the reported differences and the clinical relevance of a genetic prothrombotic score.

## Ethics Statement

This analysis was approved by the Ethics Committee of the Charité—Universitätsmedizin Berlin, Germany (EA1/048/11). As data was obtained retrospectively, consent to participate was not obtained.

## Author Contributions

KGH and BH conceived the study. KGH supervised data assessment, wrote and drafted the manuscript together with JH. JH also performed statistical analysis. BS made substantial contributions to data analysis, supervised statistical analysis, and critically revised the manuscript. BH, JK, and CN critically revised the manuscript for important intellectual content. All the authors read and approved the final manuscript.

## Conflict of Interest Statement

KGH reports lecture fees and (a) study grant(s) by Bayer Healthcare and Sanofi-Aventis, lecture fees from Boehringer Ingelheim, Pfizer, and Bristol-Myers Squibb as well as advisory board fees from Pfizer, Edwards Lifesciences, Medtronic, and EIP Pharma. CN reports lecture fees and travel support by Bayer Healthcare, Sanofi-Aventis, Bristol-Myers Squibb, Pfizer, and Boehringer Ingelheim. JK reports speaker honoraria from Bayer Healthcare Pharmaceuticals, Boehringer Ingelheim, CSL Behring, Sanofi-Aventis, Pfizer, BMS, Novartis, Aspen, Daiichi Sankyo, and Novo Nordisk. JK is also a medical advisor for CSL Behring International, Bayer HealthCare Pharmaceuticals (national and international), Astra Zeneca (national), and Novo Nordisk (national). JH, BH, and BS report no conflicts of interest.

## Abbreviations

*ACE*, angiotensin converting enzyme; *F*, factor; *F5*, factor V Leiden; *ITGA2*, glycoprotein Ia/IIa; *MTHFR*, methylenetetrahydrofolate reductase; MI, myocardial infarction; OR, odds ratio; PAOD, peripheral arterial occlusive disease; *PAI-1*, plasminogen activator inhibitor type I; *F2*, prothrombin; *FGB*, β-fibrinogen; *THBD*, thrombomodulin; *PLAT*, tissue plasminogen activator; *TFPI*, tissue-factor-pathway-inhibitor; VTE, venous thromboembolic events.
